# 

**Published:** 1892-03-19

**Authors:** 


					price one penny^
286, Vol. XI,?March 19th, 1892.
at the General Post Office as a Newspaper for
ion in the United Kingdom and Abroad J
March 19th, 1892.
WITH EXTRA NURSING SUPPLEMENT.
PerjAnnum, 6s. 6d., post?fre?.&
Monthly Parts, 6d.; post free? 8dj|
Ditto^per'Annum, 8s., post free.
No. 286.?Vol. XI.] Saturday,
March 19th, 1892.
[One Penny,

				

